# The medial circumflex femoral artery origin variability and its radiological and surgical intervention significance

**DOI:** 10.1186/s40064-015-0881-2

**Published:** 2015-03-28

**Authors:** Waseem Al-Talalwah

**Affiliations:** Department of Basic Medical Sciences, Hospital – NGHA, College of Medicine, King Abdullah International Medical Research Center / King Saud bin Abdulaziz University for Health Sciences, P.O. Box 3660, Riyadh, 11481 Saudi Arabia

**Keywords:** Medial circumflex femoral artery, Lateral circumflex femoral artery, Common femoral artery, Superficial femoral artery, Deep femoral artery

## Abstract

The medial circumflex femoral artery usually arises from the deep femoral artery. It supplies the supplies adductors and hamstring group as well as sciatic nerve and femoral head and neck through anastomosis. In current study includes 342 dissected hemipelvis to clarify the origin of medial circumflex femoral artery. The medial circumflex femoral artery arose from the common and deep femoral artery in 39.3% and 57%. Infrequently, it arose from the superficial femoral artery in 2.5% whereas it arose from the lateral circumflex femoral artery in 0.6%. In contrast, it found to be congenital absent in 0.6%. In current study, the usual origin level of medial circumflex femoral artery found to be proximal to lateral circumflex femoral artery in 52% and distal to the deep femoral artery in 57.3%. Knowing the medial circumflex femoral artery limits avascular necrosis of the femoral head such as embolization procedure. Therefore, knowing the origin variability of the medial circumflex femoral artery may lead to avoid iatrogenic fault in several procedures such as arterial bypass procedure to protect vascular supply of lower limb. Radiologists as well as orthopedics and vascular surgeons have to be aware of the medial circumflex femoral artery variation.

## Introduction

The medial circumflex femoral artery arises from medial or posterior aspect of the deep femoral artery (Carter 1867; Sharpey et al. 1867). It was known as circumflexa femoris interna (Wilson 1868) or arteriae circumflexae femoris medialis. At obturator externus, the medial circumflex femoral artery terminates into two branches are ascending and descending (Carter 1867), anterior and posterior (Sharpey et al. 1867), muscular and articular (Wilson 1868), and superficial and deep (Standring 2005). Medial circumflex femoral artery supplies adductors, gracillis, obturator externus and hamstring muscle (Carter 1867; Sharpey et al. 1867; Wilson 1868). It also supplies the sciatic nerve (Georgakis 2008) It anastomoses with the inferior gluteal, lateral circumflex femoral and first perforating arteries (Carter 1867; Sharpey et al. 1867; Wilson 1868) refereed as cruciate anastomosis supplying the head and neck of the femur (Moore & Persaud 1998; Williams et al. 1989). Furthermore, the medial circumflex femoral artery is a chief artery in vascularization of head and neck femur (Oide 1979; Clarke & Colborn 1993). Variability of medial circumflex femoral artery is due to primitive plexus (Moore & Persaud 1998) during development and regression of primary axial artery (Sidway 2005; Kalhor et al. 2009) result in different supply of the lower extremity (Moore & Persaud 1998; Lippert & Pabst 1985; Perera 1995).

The current study targets the origin of medial circumflex femoral artery and its level. With a good background of the anatomical characteristics of medial circumflex femoral artery, it may reflect a clinical important in radiology, vascular surgery and orthopedic fields to minimize the postsurgical complications.

## Materials and method

The present study includes 342 hemipelves from 171 cadavers were dissected to study the medial circumflex femoral artery origin and its branches. This study is conducted in centre for anatomy and human identification, college of life science, University of Dundee. The entire specimens have been dissected the photos have been taken by the author under permission and regulation of United Kingdom. The missing data has been excluded to provide the accurate incidence of the variation. The origin variability of the medial circumflex femoral artery and its level compare to the deep femoral and lateral circumflex femoral arteries have been described to provide sufficient data for radiologist in femoral catheterization.

Previous to the anterior and medial compartment dissection, the anterior superior iliac spine and pupic tubercle has to be identified by deep palpation. An oblique incision has to be below the inguinal ligament ends to clarify anterior compartment. Then, the detachment is including skin, membranous and fatty layer has to be removed. A great carful during removing subcutaneous tissue is due to great saphenous course. The great saphenous vein pass over the medial side and penetrate a defect of the deep fascia known as fossa ovalis where the lateral margin called falciform margin to drain into femoral vein. After that, the deep fascia should be removed as soon as the anterior compartment muscle could be inspected. The anterior compartment includes the quadriceps femoris, Sartorius pectineus, iliacus and iliopsoas (psoas major and minor). The quadriceps femoris have four heads are: rectus femoris, vastus lateralis, vastus medialis and vastus intermedius. Organizations of these previous muscles give a triangle known as femoral triangle which is formed by inguinal ligament superiorly, Sartorius laterally and adductor longus medially. The latter muscle forms the floor of this triangle partially and completed by iliopsoas and pectineus. The femoral triangle contains from lateral to medial is femoral nerve, femoral artery, femoral vein and femoral ring (contains a lymph node). The deep fascia of the abdominal wall expansion refers as femoral sheath which contains last three structures forming three compartments. The medial, the intermediate and lateral compartments conclude femoral canal, femoral vein and femoral artery correspondingly. A femoral vein system has to be inspected in relation to femoral artery with awareness before removing time to clarify the femoral artery and its branches. The femoral artery starts just below the inguinal ligament as a continuation of the external iliac artery and terminates as popliteal artery at the adductor (Hunter) hiatus. The femoral artery is known as common femoral artery by radiologist. Therefore, the common femoral artery bifurcates into superficial and deep femoral (profunda femoris) arteries. So, the superficial femoral artery is a segment starting from site of the femoral artery ending at adductor hiatus as popliteal artery. The superficial femoral artery has to be traced till termination as popliteal artery. The typical bifurcation of femoral artery is into superficial and deep femoral artery as the profunda femoris artery usually gives medial and lateral femoral circumflex arteries. The medial femoral circumflex branch runs medially and posteriorly between pectineus and iliopsoas and divides in to anterior and posterior branch. To clarify this artery, the femoral vein and its trabiturates has to be got rid of it. During medial femoral circumflex vein remove, a great attention has to be paid to avoid unnecessary extraction of medial femoral circumflex branch. The medial femoral circumflex branch is a standard branch of profunda femoris artery but a possibility of this branch arise independently or dependently (same trunk) from the common femoral artery and superficial femoral artery. Furthermore, medial circumflex femoral artery may arise with the superficial and deep femoral artery arteries. It may also arise with the previous two arteries and lateral circumflex femoral artery.

## Result

**Figure 1:**
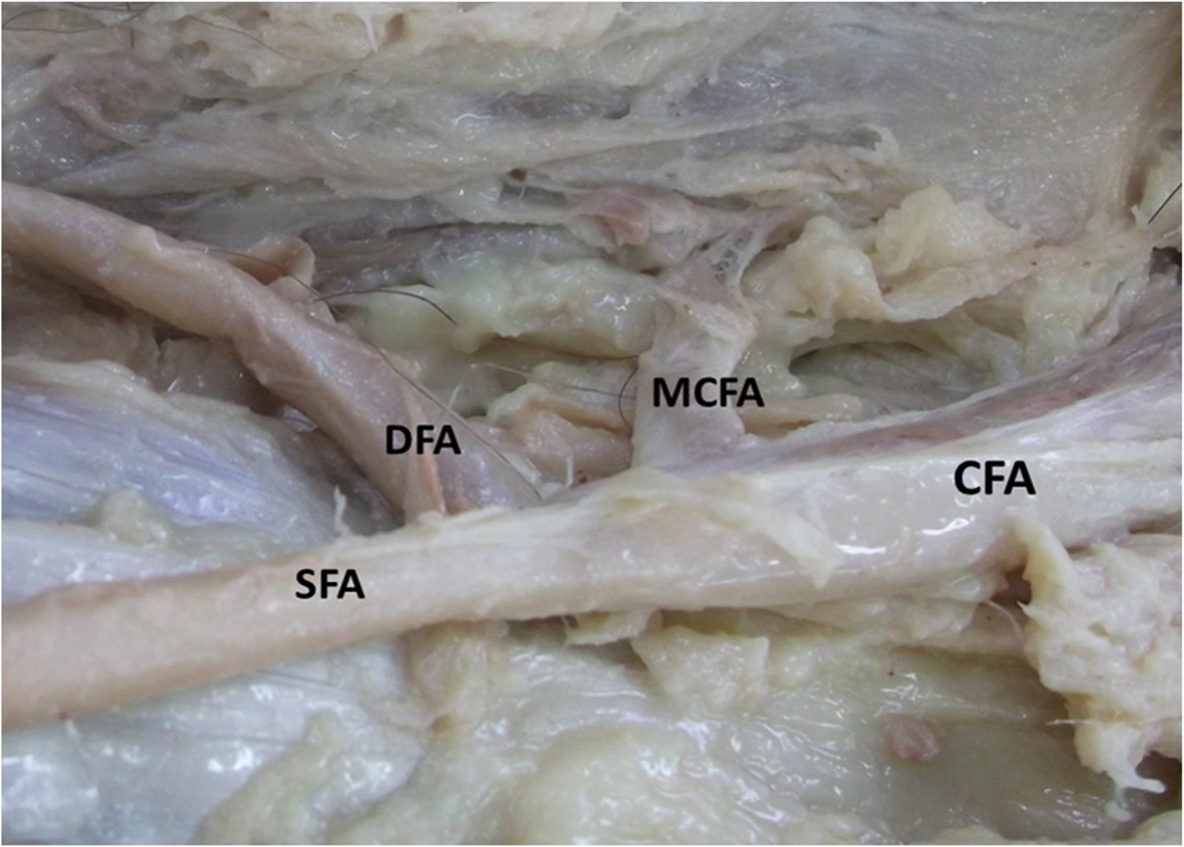
**The medial circumflex femoral artery arising from the common femoral artery.** CFA. Common femoral artery, SFA. Superficial femoral artery, DFA. Deep femoral artery, MCFA. Medial circumflex femoral artery, LCFA. Lateral circumflex femoral artery.

**Figure 2:**
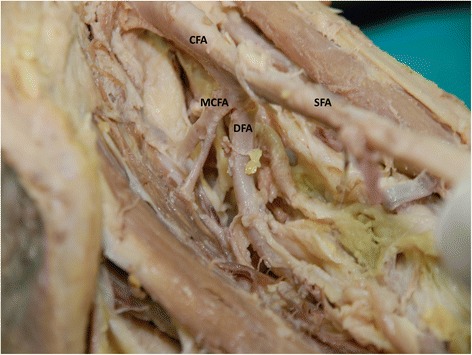
**The medial circumflex femoral artery arising from the common trunk of femoral artery with the deep femoral artery.** CFA. Common femoral artery, SFA. Superficial femoral artery, DFA. Deep femoral artery, MCFA. Medial circumflex femoral artery.

**Figure 3:**
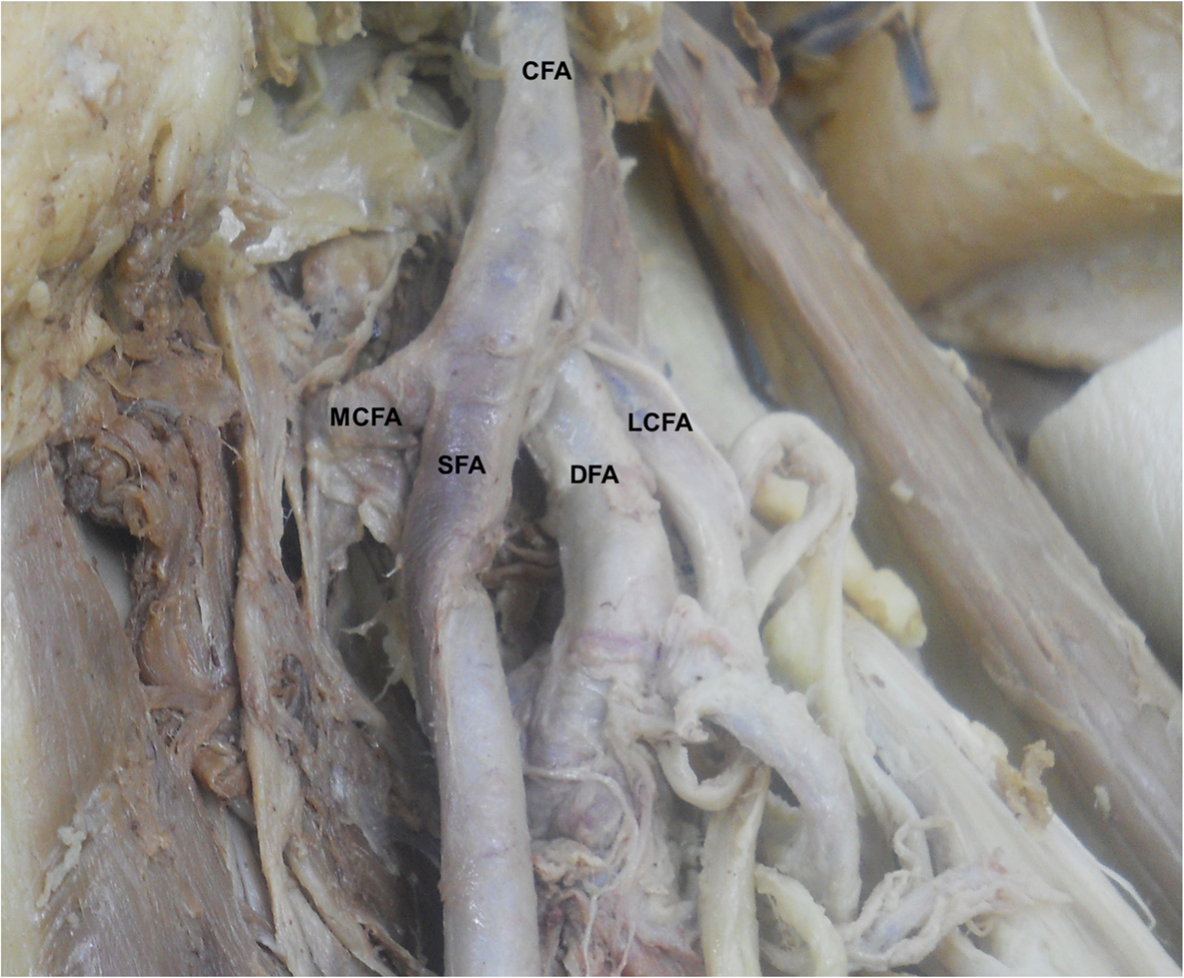
**The medial circumflex femoral artery arising from the common trunk of femoral artery with the superficial and deep femoral arteries as well as the lateral circumflex femoral artery.** CFA. Common femoral artery, SFA. Superficial femoral artery, DFA. Deep femoral artery, MCFA. Medial circumflex femoral artery, LCFA. Lateral circumflex femoral artery.

**Figure 4:**
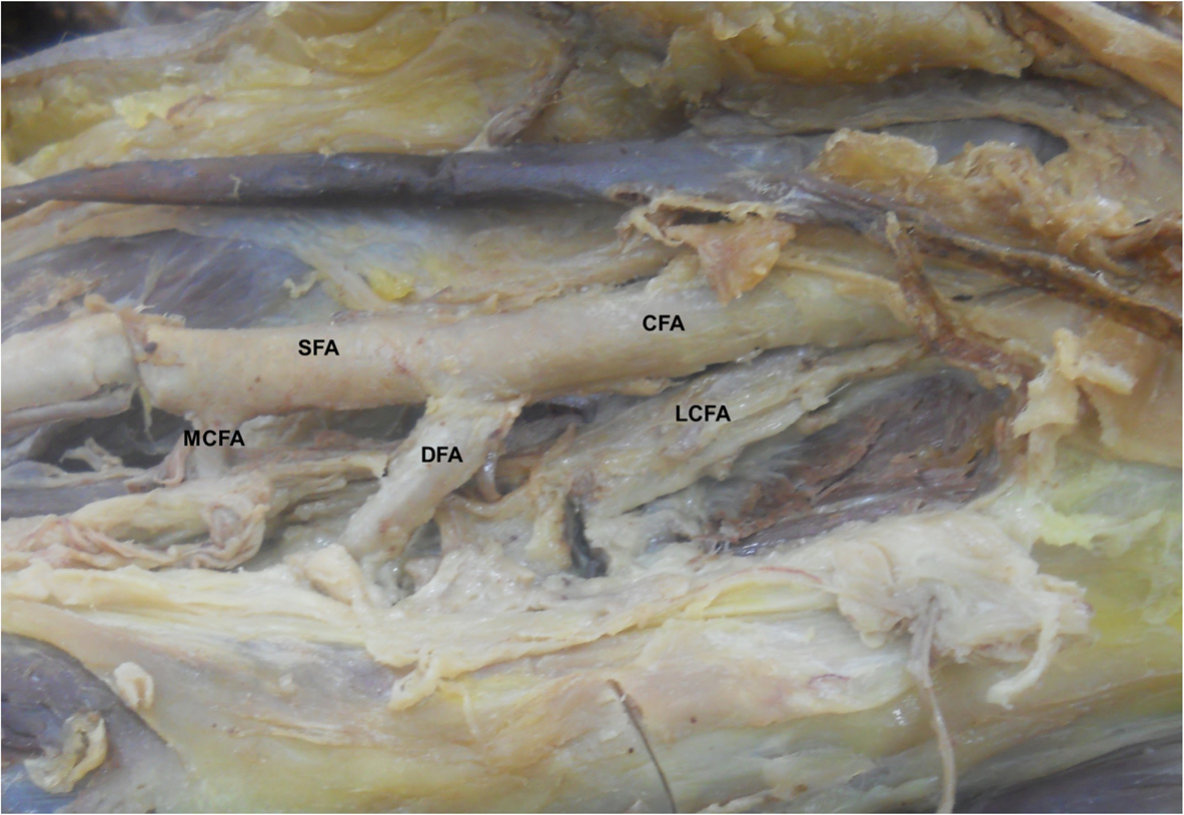
**The medial circumflex femoral artery arising from the superficial femoral artery.** CFA. Common femoral artery, SFA. Superficial femoral artery, DFA. Deep femoral artery, MCFA. Medial circumflex femoral artery, LCFA. Lateral circumflex femoral artery.

**Figure 5:**
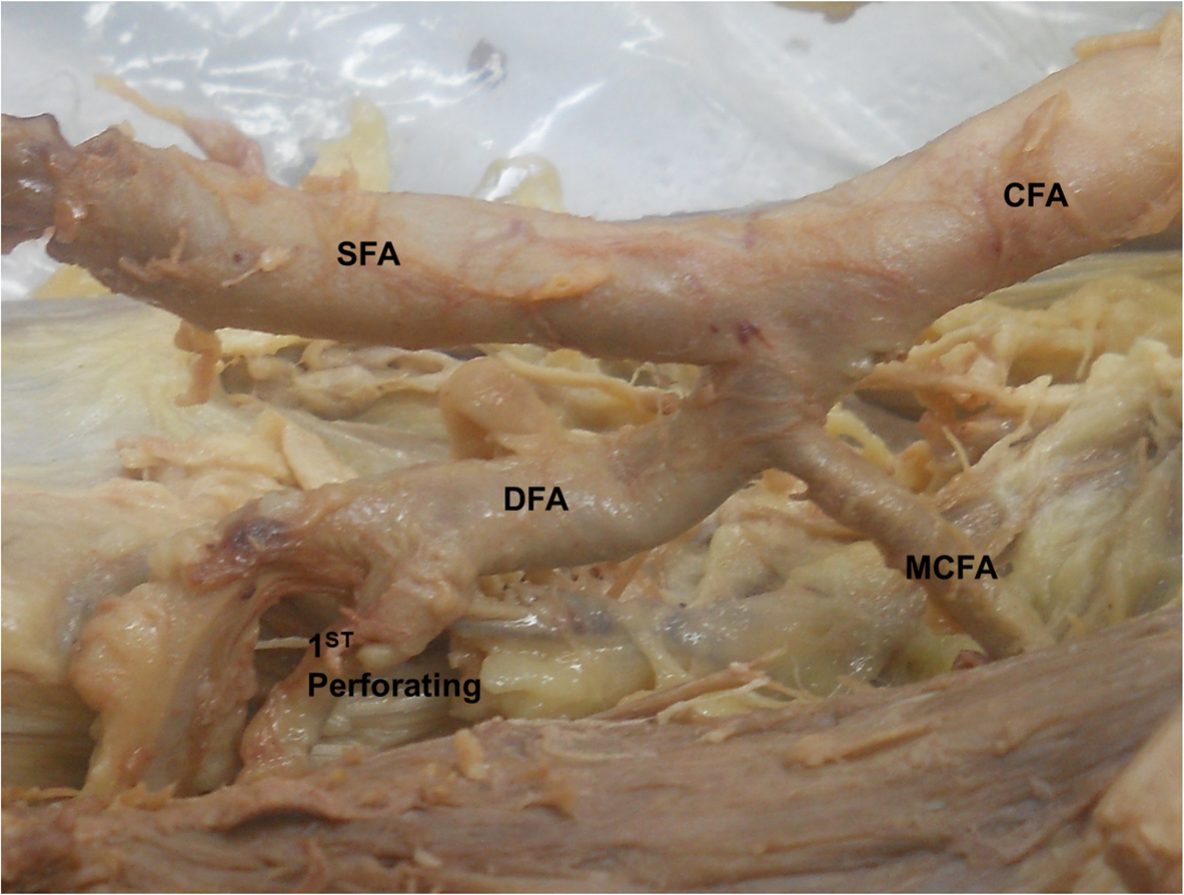
**The medial circumflex femoral artery arising from the deep femoral artery.** CFA. Common femoral artery, SFA. Superficial femoral artery, DFA. Deep femoral artery, MCFA. Medial circumflex femoral artery, 1^st^ perforating. First perforating artery.

**Figure 6:**
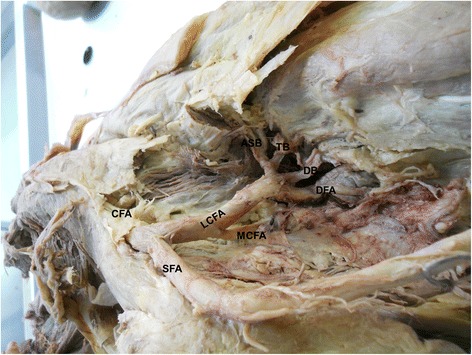
**The medial circumflex femoral artery arising from the lateral circumflex femoral artery.** CFA. Common femoral artery, SFA. Superficial femoral artery, DFA. Deep femoral artery, MCFA. Medial circumflex femoral artery, LCFA. Lateral circumflex femoral artery, ASB. Ascending branch, TB. Transverse branch, DB. Descending branch.

In present study, the medial circumflex femoral artery arises from the common femoral artery in 39.3% (Figure 1). It arose from common femoral artery independently in 13.1% (Figure 1) and dependently with deep femoral artery (Figure 2) or with lateral circumflex femoral artery in 14.6% or in 1.9%. It also found to be arising from the common femoral artery with superficial and deep femoral artery and lateral circumflex femoral artery in 9% (Figure 3). In few cases, the medial circumflex femoral artery arises from common femoral artery with external pudendal artery in 0.7%. As the common femoral artery bifurcates into superficial and deep femoral arteries, it arises from the superficial and deep femoral arteries in 2.5% and in 57% respectively (Figure 4). The medial circumflex femoral artery arises from the deep femoral artery independently 50.2% (Figure 5) and dependently as with the lateral circumflex femoral artery in 6.8%. In few cases, the medial circumflex femoral artery arises from the lateral circumflex femoral artery in 0.6% (Figure 6). It found to be a congenital absence in 0.6% (Table 1).

**Table 1:** **The variable origin of medial circumflex artery**

**Origin**	**Incidence %**
**CFA**	39.3
**CFA Independently**	13.1
**CFA Dependently**	
**Femoral trunk--- with DFA**	14.6
**Femoral trunk ---with LCFA**	1.9
**Femoral trunk--- with DFA and LCFA**	9
**Femoral trunk--- with EPA**	0.7
**SFA**	2.5
**DFA**	57
**DFA independently**	50.2
**DFA dependently**	
**DFA Trunk With LCFA**	6.8
**LCFA**	0.6
**CAB**	0.6

Current study includes 342 specimens and investigates the origin of medial circumflex femoral artery (MCFA) from Common femoral artery (CFA), superficial femoral artery (SFA), Deep femoral artery (DFA) and Lateral circumflex femoral artery (LCFA). It arise independently (Directly) or independently (Indirectly) as from trunk with other artery such as External pudendal artery (EPA). It found to be congenital absence (CAB).

The origin level of the medial circumflex femoral artery in relation to the origin level of the deep femoral artery is inconstant. The medial circumflex femoral artery arises proximal and distal to the deep femoral artery origin in 16.8% and 57.3% respectively (Figures 1,2,3 and 4). However, the origins of both arteries have same level in 25.2%. In case of congenital absence of the medial circumflex femoral artery, the origin level of the medial circumflex femoral artery in relation to the origin level of the deep femoral artery is difficult to compare in 0.6% (Table 2). The origin level of the medial circumflex femoral artery in relation to the origin level of the lateral circumflex femoral artery is variable. The medial circumflex femoral artery arises proximal and distal to the lateral circumflex femoral artery origin in 52% and 24% respectively (Figures 4 and 6). However, the origins of both arteries have same level in 22.8%. In case of congenital absence of the medial circumflex femoral artery, the origin level of the medial circumflex femoral artery in relation to the origin level of the lateral circumflex femoral artery is difficult to compare in 1.2% (Table 3).

**Table 2:** **The origin level of Medial circumflex femoral artery in relation to deep femoral artery**

**Relation**	**Incidence %**
**Proximal**	16.8
**Same**	25.2
**Distal**	57.3
**Irrelevant**	0.6

Current study includes 342 specimens (158 Female and 184 male thigh regions). (F). Current study includes 342 specimens and investigates the origin level of medial circumflex femoral artery (MCFA) in relation to deep femoral artery origin level. It found to be congenital absence (CAB) therefore it is hard to compare (Irrelevant).

**Table 3:** **The origin level of Medial circumflex femoral artery in relation to lateral circumflex femoral artery**

**Relation**	**Incidence %**
**Proximal**	52
**Same**	22.8
**Distal**	24
**Irrelevant**	1.2

Current study includes 342 specimens (158 Female and 184 male thigh regions). (F). Current study includes 342 specimens and investigates the origin level of medial circumflex femoral artery (MCFA) in relation to lateral circumflex femoral artery (LCFA) origin level. It found to be congenital absence (CAB) therefore it is hard to compare (Irrelevant).

## Discussion

Embryologically, the primary axial artery is a chief artery of lower limb. During lower limb development, new vessels develop and distribute in bud during 3 months McClellan (Moore & Persaud 1998). Based on sideway (2005) theory (Kalhor et al. 2009), the femoral system develop as the sciatic artery regress. The medial circumflex femoral artery developed an independently from the rete femorale as a result of the blood flow projected in unusual region leading to unusual choice of source channels. This could explain the unusual origin site of the medial circumflex femoral artery arising from posteriolateral in-stead of posteriomedial aspect of the femoral artery usually (Çiftcioglu et al. 2009). Therefore, anatomical variation results in diverse supply of the lower extremity (Moore & Persaud 1998; Lippert & Pabst 1985; Perera 1995). Therefore, the variability of the medial circumflex femoral artery in origin and its level in relation to deep femoral and medial circumflex femoral arteries is due embryologic development of the primitive plexus of femoral trees and the primitive axial artery regression either completely or incompletely.

The origin variability of the medial circumflex femoral artery divides into two groups. The first group, the medial circumflex femoral arteries originated from the deep femoral artery. The second group, the medial circumflex femoral arteries originated from the common femoral artery (Perera 1995) (Figure A). According to series studies, the medial circumflex femoral artery arose from the deep femoral artery in different incidence ranging from 12% to 85.7% (Clarke & Colborn 1993; Lippert & Pabst 1985; Quain 1844; Srb 1860; Auburtin 1905; Lipshutz 1918; Adachi 1928; Charles et al. 1930; Williams et al. 1934; Suder & Nizankowski 1935; Ming-Tzu 1937; Chand & Singh 1951; Keen 1961; Videau et al. 1964; Gremigni 1968; Leborgne et al. 1974; Marcade et al. 1978; Guillot et al. 1979; Siddharth et al. 1985; Emura et al. 1989; Massoud & Fletcher 1997; Gautier et al. 2000; Dixit et al. 2001; Başar et al. 2002; Tanyeli et al. 2006; Vazquez et al. 2007; Samarawickrama et al. 2009; Prakash et al. 2010; Dixita et al. 2011; Lalović et al. 2013; Peera & Sugavasi 2013; Shiny Vinila et al. 2013) (Table 4). In present study, the medial circumflex femoral artery arose from the deep femoral artery in 57% (Table 1). However, the medial circumflex femoral artery arose from the femoral artery with different incidences ranging from 11% to 78% (Clarke & Colborn 1993; Quain 1844; Srb 1860; Auburtin 1905; Lipshutz 1918; Adachi 1928; Charles et al. 1930; Williams et al. 1934; Suder & Nizankowski 1935; Ming-Tzu 1937; Chand & Singh 1951; Keen 1961; Videau et al. 1964; Gremigni 1968; Leborgne et al. 1974; Marcade et al. 1978; Guillot et al. 1979; Siddharth et al. 1985; Emura et al. 1989; Massoud & Fletcher 1997; Gautier et al. 2000; Dixit et al. 2001; Başar et al. 2002; Tanyeli et al. 2006; Vazquez et al. 2007; Samarawickrama et al. 2009; Prakash et al. 2010; Dixita et al. 2011; Lalović et al. 2013; Peera & Sugavasi 2013; Shiny Vinila et al. 2013; Colborn et al. 1995) (Table 4). In present study, the medial circumflex femoral artery arose from the femoral artery in 39.3% (Table 1).

**Table 4:** **The incidence of variable origin of medial circumflex femoral artery in series study**

**Study**	**Incidence %**	
	**Common femoral artery**	**Deep femoral artery**
**Quain (** 1844 **)**	45.6	54.3
**Srb (** 1860 **)**	61.2	38.8
**Auburtin (** 1905 **)**	62.5	37.5
**Lipshutz (** 1918 **)**	43.2	56.8
**Adachi (** 1928 **)**	50.9	49.1
**Charles et al (** 1930 **)**	65	35
**Williams et al (** 1934 **)**	61.4	38.6
**Suder and Nizankowski (** 1935 **)**	21	79
**Ming-Tzu (** 1937 **)**	54.5	45.4
**Chand and Singh (** 1951 **)**	69.6	30.4
**Keen (** 1961 **)**	60.8	39.2
**Videau et al (** 1964 **)**	63	37
**Gremigni (** 1968 **)**	22	12
**Leborgne (** 1974 **)**	77.8	22.2
**Marcade et al (** 1978 **)**	14.3	85.7
**Guillot et al (** 1979 **)**	70	30
**Lippert and Pabst (** 1985 **)**	None*	58
**Siddharth et al (** 1985 **)**	26	63
**Suder and Nizankowski (** 1985 **)**	21	None*
**Emura et al (** 1989 **)**	11.6	61.7
**Clarke and Colborn (** 1993 **)**	40	53
**Colborn et al (** 1995 **)**	25	None*
**Massoud and Fletcher (** 1997 **)**	18	81
**Gautier et al (** 2000 **)**	16.7	83.3
**Dixit et al (** 2001 **)**	20.63	62.5
**Basar et al (** 2002 **)**	48.9	51.1
**Tanyeli et al (** 2006 **)**	15	79
**Vazquez et al (** 2007 **)**	77.8	22.2
**Samarawickrama (** 2009 **)**	31	62
**Prakash et al (** 2010 **)**	32.8	67.2
**Dixita et al (** 2011 **)**	38.6	61.4
**Lalović et al (** 2013 **)**	33.3	59.5
**Peera and Sugavasi (** 2013 **)**	20	75
**Shiny Vinila et al (** 2013 **)**	18.4	65

*None is not mentioned in the study.

Based on series study as well as current study, the origin of the medial circumflex femoral artery is more commonly from the deep femoral artery (Clarke & Colborn 1993; Quain 1844; Lipshutz 1918; Suder & Nizankowski 1935; Marcade et al. 1978; Siddharth et al. 1985; Emura et al. 1989; Massoud & Fletcher 1997; Gautier et al. 2000; Dixit et al. 2001; Başar et al. 2002; Tanyeli et al. 2006; Samarawickrama et al. 2009; Prakash et al. 2010; Dixita et al. 2011; Lalović et al. 2013; Peera & Sugavasi 2013; Shiny Vinila et al. 2013). Whereas in other studies, the origin of the medial circumflex femoral artery is more commonly from the femoral artery (Srb 1860; Auburtin 1905; Adachi 1928; Charles et al. 1930; Williams et al. 1934; Ming-Tzu 1937; Chand & Singh 1951; Keen 1961; Videau et al. 1964; Gremigni 1968; Leborgne et al. 1974; Guillot et al. 1979; Vazquez et al. 2007; Colborn et al. 1995) (Table 4). Therefore, the incidence medial circumflex femoral artery arises from either the common or deep femoral artery is inconstant.

The medial circumflex femoral artery arises from superficial femoral artery 6.7% reported by Dixita et al. (2011). Also, this variation found to be in 2.5% in current study. Recently, it arising from lateral circumflex femoral artery in 15% has been reported by Peera and Sugavasi^44^ but it arises in 0.6% in current study. Series studies classified the origin variability of the medial circumflex femoral artery based on its arising independently or dependently with other artery in different types (Emura et al. 1989; Tanyeli et al. 2006; Vazquez et al. 2007). In present study, it arises independently as from the common, superficial and deep femoral arteries or dependently from a trunk of the common and deep femoral arteries with external pudendal, lateral circumflex or deep femoral arteries.

The medial circumflex femoral artery arises from common trunk of femoral artery with deep femoral artery occurring in 4% (Adachi 1928), in 1% (Tanyeli et al. 2006), in 15.4% (Dixita et al. 2011), in 2.4% (Lalović et al. 2013) or in 5% (Peera & Sugavasi 2013) and in 14.6% in current study (Table 1). The medial circumflex femoral artery may arise from a trunk of femoral artery with the lateral circumflex femoral and deep femoral artery in in 5% (Çiftcioglu et al. 2009; Siddharth et al. 1985), in 1% (Tanyeli et al. 2006), in 2.5% (Baptist et al. 2007), in 8% (Samarawickrama et al. 2009) or in 17.5% (Shiny Vinila et al. 2013). In this study, it is occurred in 9% (Table 1). The medial circumflex femoral artery may arise from a common trunk of femoral artery with deep external pudendal artery 11.6% (Samarawickrama et al. 2009) or 17.7% (Shiny Vinila et al. 2013). In present study, it is very rare variation occurred in 0.6% (Table 1). The medial circumflex artery arises from the common trunk of the deep femoral artery with different arteries in 20.88% (Dixit et al. 2001). In current study, it arises from a trunk of deep femoral artery with other artery in 6.8% (Table 1).

In general, the incidence of origin variability of the medial circumflex femoral artery in current study is differ from the previous study may due to several factors such as races and genetics which responsible of different patterns femoral systems as well as its incidences in different population.

The origin levels of the medial circumflex femoral artery in relation to lateral circumflex femoral arteries arising from deep femoral artery has been classified into three forms by (Dixita et al. (2011). The first form, the medial circumflex femoral artery arises proximal to the origin of the deep femoral artery in 16.7%. The second form, the medial circumflex femoral artery arises distal to the origin of the deep femoral artery in 6.7%. The third form, it has a same origin level of the deep femoral artery in 15.4%. In current study, the medial circumflex femoral artery arises proximal and distal to the origin of the deep femoral artery in 16.8% and in 57.3% whereas it has a same origin level of the deep femoral artery in 25.2% (Table 2). The origin levels of the medial circumflex femoral artery in relation to lateral circumflex femoral arteries arising from deep femoral artery has been classified into three forms by Vazquez et al (Vazquez et al. 2007). First form is the medial circumflex femoral artery arising proximal to the lateral circumflex femoral artery origin in 53.2% while in 52% in current study. The second form is medial circumflex femoral artery arising distal to the lateral circumflex femoral artery origin in 23.4% while in 24% in current study. The third form is the both circumflex femoral arteries originating from same level as from common trunk of deep femoral artery in 23.4% while in 6.9% in current study (Table 3).

In current study, the medial circumflex femoral artery origin has been classified into three forms. First form, the medial circumflex femora artery arises from common femoral artery. Second form, the medial circumflex femora artery arises from superficial femoral artery. Third form, the medial circumflex femora artery arises from deep femoral artery. Each form subdivided into subdivision: Independent (Direct) and dependent (Indirect) type as from the main artery and a trunk with other artery respectively. Several variations of the medial circumflex artery exist based on number on each side. For instance, a double medial circumflex artery on each side found to be in four cases with (4%) (Tanyeli et al. 2006). On the other hand, the congenital absence of the medial circumflex femoral artery found to be 0.3% by Vazquez et al (Vazquez et al. 2007). Recently, it has reported to be congenital absence in 4.8% (Lalović et al. 2013). In current study, the medial circumflex femoral artery is congenital absent in 0.6% (Table 1).

Knowing the medial circumflex femoral artery limits avascular necrosis of the femoral head such as embolization procedure. Therefore, radiologists have to be aware of the medial circumflex femoral artery origin to report orthopedics and vascular surgeons leading to reduce the iatrogenic fault. Further, this study may help in end to end arterial (anastomosis) bypass procedure to preserve vascular supply of lower limb.

## Conclusion

Understanding anatomical feature of the medial circumflex femoral artery may help in decreasing incidence of avascular necrosis of the femoral head during embolization, arterial catheterization procedure or hip surgery (Oide 1979; Kalhor et al. 2009; Güttler et al. 2007). Therefore, radiologists have to be aware of the medial circumflex femoral artery origin to alert vascular and orthopedic surgeons to diminish the iatrogenic error. Consequently, the variable origin of medial circumflex femoral artery and its level is clinically important to modify the end to end arterial (anastomosis) bypass procedure or interposition graft operation leading to intact vascular supply of lower extremities.
